# Validating linkage of multiple population-based administrative databases in Brazil

**DOI:** 10.1371/journal.pone.0214050

**Published:** 2019-03-28

**Authors:** Enny S. Paixão, Oona M. R. Campbell, Laura C. Rodrigues, Maria Glória Teixeira, Maria da Conceição N. Costa, Elizabeth B. Brickley, Katie Harron

**Affiliations:** 1 London School of Hygiene and Tropical Medicine, Bloomsbury, London, United Kingdom; 2 Instituto de Saúde Coletiva, Rua Basílio da Gama, s/n.Canela, CEP, Salvador, Bahia, Brazil; University of South Wales, AUSTRALIA

## Abstract

**Background:**

Linking routinely-collected data provides an opportunity to measure the effects of exposures that occur before birth on maternal, fetal and infant outcomes. High quality linkage is a prerequisite for producing reliable results, and there are specific challenges in mother-baby linkage. Using population-based administrative databases from Brazil, this study aimed to estimate the accuracy of linkage between maternal deaths and birth outcomes and dengue notifications, and to identify potential sources of bias when assessing the risk of maternal death due to dengue in pregnancy.

**Methods:**

We identified women with dengue during pregnancy in a previously linked dataset of dengue notifications in women who had experienced a live birth or stillbirth during 2007–2012. We then linked this dataset with maternal death records probabilistically using maternal name, age and municipality. We estimated the accuracy of the linkage, and examined the characteristics of false-matches and missed-matches to identify any sources of bias.

**Results:**

Of the 10,259 maternal deaths recorded in 2007–2012, 6717 were linked: 5444 to a live birth record, 1306 to a stillbirth record, and 33 to both a live and stillbirth record. After identifying 2620 missed-matches and 124 false-matches, our estimated sensitivity was 72%, specificity was 88%, and positive predictive value was 98%. Linkage errors were associated with maternal education and self-identified race; women with more than 7 years of education or who self-declared as Caucasian were more likely to link. Dengue status was not associated with linkage error.

**Conclusion:**

Despite not having unique identifiers to link mothers and birth outcomes, we demonstrated a high standard of linkage, with sensitivity and specificity values comparable to previous literature. Although there were no differences in the characteristics of dengue cases missed or included in our linked dataset, linkage error occurred disproportionally by some social-demographic characteristics, which should be taken into account in future analyses.

## Background

Research involving record linkage has increased in recent years with the growing availability of administrative population-based databases and the relatively low cost of joining information from different sources. Linkage has been particularly important for research on maternal, fetal and infant health. Maternal mortality in Brazil is still relatively high, at 62 maternal deaths per 100,000 live births in 2015. Therefore, studying potential risk factors associated with this health indicator is a priority in the public health agenda[1].

Linking information on women’s social contexts and health status prior to and during pregnancy to fetal and childhood outcomes can yield knowledge beyond the limits of traditional cohort studies. Due to the large scale and availability of population-based datasets, and the routine collection of such data (i.e., they do not require a large cohort with many years of follow-up to be established), rare or long-term outcomes in particular can be investigated. In the past decade, many high-income countries have used linkage to identify maternal deaths and to investigate the health of mothers and their babies[2–5]. However, for low and middle income countries, record linkage methods have only been developed more recently[6].

The quality of the linkage is important for producing reliable results, and there are specific challenges in mother-baby linkage, mainly arising from to the limited availability of common identifiers for linkage [1]. The impact of linkage error depends on the question of interest[7]. For example, linkages with low sensitivity cannot be used to measure the incidence of a given disease, such as cancer, in a population, because the measure calculated will be underestimated [8]. However if the study presents a low sensitivity randomly distributed between groups, the measure of association will be unbiased, so it can still be useful under this circumstance, as shown by a study of dengue during pregnancy and stillbirth[6]. In other research, it may be more important to prioritise specificity over sensitivity, mainly because low specificity will overestimate rare outcomes. For example, in case-control studies, we may be more concerned with precisely establishing exposure status through linkage, rather than capturing all links[6]. In this case, it is important to avoid selection bias (if missed-matches are non-random, i.e. are more likely to occur in specific subgroups of records).

This linkage study is part of a series of studies measuring the association between dengue and pregnancy outcomes in Brazil[6,9]. Brazil has universal health coverage (including emergency care for tourists and illegal immigrants) and all births and deaths must be notified. It also has a list of diseases that are compulsory to notify, including dengue. Brazil has a long tradition of collecting administrative routine data and therefore meets the criteria for conducting linkage studies.

In this article, we aimed to validate the linkage strategy we used in Paixao et al 2017[6] to create a cohort study on the effects of dengue on maternal and birth outcomes. In this, linkage was required to establish exposure status. Reports of negative pregnancy outcomes after dengue infection are not new. However, evidence for the association between maternal dengue and adverse pregnancy outcomes is limited and controversial, and there are few population-based studies that estimate the magnitude of the risk while appropriately controlling for confounding and with enough observations to study rare outcomes such as maternal deaths. The outcome of these series of studies will enable physicians to properly conduct a risk assessment in pregnant patients and policy-makers to use this information to include women with dengue in pregnancy in an “at risk” population.

Evaluating a linkage strategy with a dataset (a maternal mortality dataset) for which we have *a priori* information on the expected number of matches gives us information about the likely quality of the same linkage strategy when applied to datasets where the expected number of matches is not known (linkage between live births, stillbirths and dengue notifications). The purpose of our current analysis (linkage of maternal death records with live births and stillbirths in the context of maternal dengue infections) was to investigate whether any linkage errors were differently distributed amongst the group with the outcome (maternal deaths) and the comparison group (women who survived). This would establish any potential bias in the linked dataset, which might influence subsequent analyses. We aimed to estimate the accuracy of the linkage and identify potential sources of bias, where any subgroups of records were more or less likely to link.

## Methods

### Datasets

**Fig 1:**
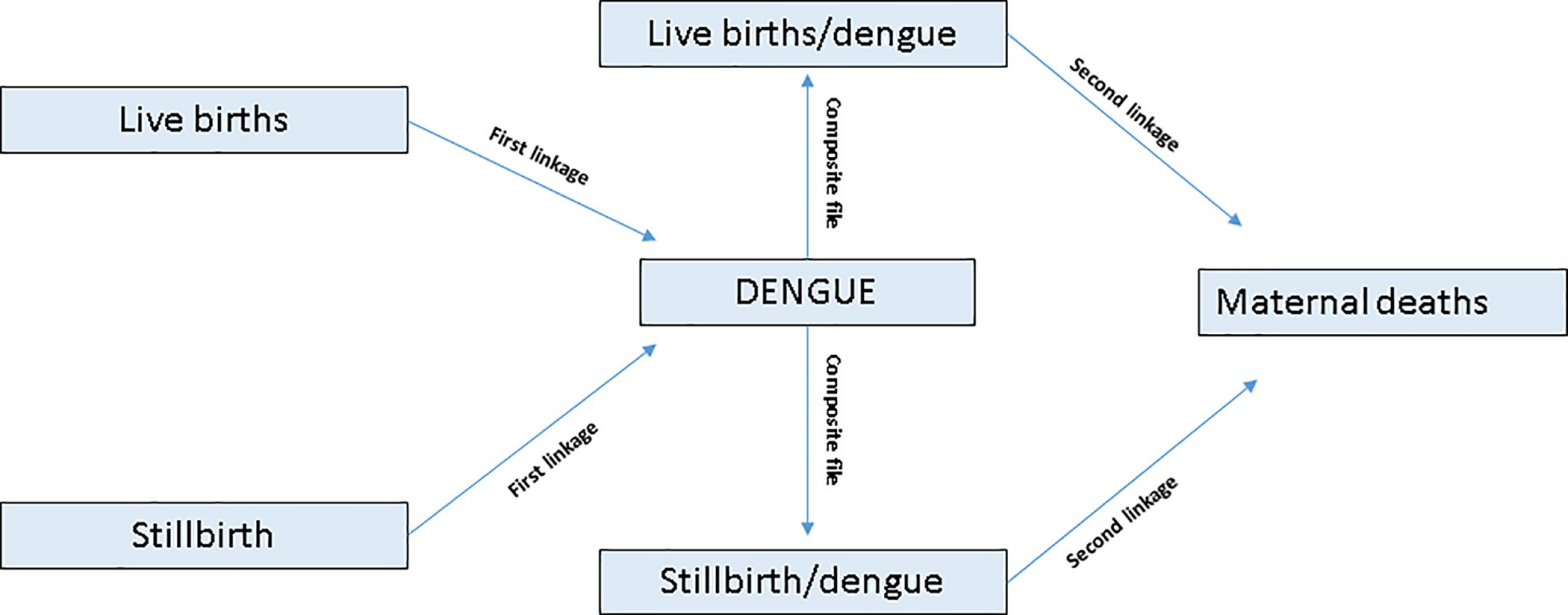
Linkage strategy diagram.

We linked three routinely collected Brazilian datasets: a) Notifiable Diseases Information (Sistema de Informação de Agravos de Notificação; SINAN) for dengue; b) Live Births Information System (Sistema de Informação sobre Nascimentos; SINASC) for live births; and c) Mortality Information System (Sistema de Informação sobre Mortalidade;SIM) for stillbirths and maternal deaths. We linked the datasets in two stages (Fig 1). First, we probabilistically linked dengue notifications in women (SINAN) with records of women who had live births (SINASC) or stillbirths (SIM), to identify those women who had dengue during pregnancy during 2007–2012. This first linkage (between women with dengue notifications and with live births/stillbirths) is described in detail elsewhere[6]. Second, we linked this composite file (women with a dengue notification linked with live births/stillbirths) with maternal death records for 2007–2012. This paper focusses on the results of this second stage of the linkage.

In both linkage processes, we used variables common in all three datasets: maternal name, maternal age, and place of residence. To link with death records, we also used information on the date of the maternal death and the birth outcome, because maternal deaths are likely to occur on the same day as the birth outcome or soon after (and by definition must occur within 42 days after the end of pregnancy). Therefore, the difference in dates contributed information on the likelihood of two records being a match.

### Data Source 1: Brazilian Notifiable Diseases Information System -SINAN

The Brazilian Notifiable Diseases Information System (SINAN) contains records on notifiable diseases, characteristics of the individual with the disease (name, place of residence, age, sex, and years of education), symptoms of the disease, laboratory tests, and disease severity.

After excluding men, and non-dengue records, we retained 1,725,943 cases of dengue from 2007–2012. This is likely to underestimate the burden of disease, as less than 10% of dengue cases are reported to SINAN in Brazil. For every notified case, there are an estimated 12 dengue cases in the community[10].

### Data Source 2: Brazilian Live Birth Information System -SINASC

The Ministry of Health of Brazil uses the WHO definition of a live birth: the complete expulsion or extraction from the body of the pregnant woman of a product of conception, independent of the duration of pregnancy, who, after the separation, breathes or shows any other signs of life, such as heartbeat, umbilical cord pulsation, or definite movement of voluntary muscles, whether or not the cord is cut and whether or not the placenta is attached. The Brazilian Live Birth Information System (SINASC) contains records of all live births in Brazil; these data come from birth registration, a legal document completed by the health worker who attends the birth. It includes information on the mother (name, place of residence, age, marital status, education); the pregnancy (length of gestation, type of delivery); and the neonate (birth-weight, the presence of congenital anomalies). An evaluation of the birth registration system in Brazil found 97% of Brazilian live births are registered.

### Data Source 3: Brazilian Mortality Information System -SIM

The Ministry of Health of Brazil uses the WHO definition of maternal death: the death of a woman while pregnant or within 42 days of termination of pregnancy, irrespective of the duration and site of the pregnancy, from any cause related to or aggravated by the pregnancy or its management but not from accidental or incidental causes. Maternal mortality is widely underreported worldwide. Since 2008, the health surveillance system in Brazil has investigated deaths in women of 15–49 to enhance detection of maternal deaths. In 2010, 76% of deaths occurred in this group were investigated[1].

The Brazilian Mortality Information System (SIM) contains records of all deaths in Brazil, including stillbirths. Stillbirth in Brazil is defined as the death of a product of conception before the expulsion or complete extraction from the body of the pregnant woman, occurring from 22 weeks or weighing more than 500g). These data come from the Death Certificate, a required legal document. We retained all maternal deaths, defined as those coded under obstetric causes of death by ICD-10, the "O" group), and all stillbirths.

It is possible that stillbirths are under-reported in the national system[11], however, it is difficult to estimate the extent of under-ascertainment. After excluding records without a mother’s name, we retained 187,487 stillbirth records and 10,259 maternal deaths (with an obstetric code in ICD-10 recorded as the cause of death) extracted from SIM (2007–2012).

### Completeness of linkage variables

Table 1 contains information on the number of missing values in the variables used in the linkage process in the three datasets (SIM, SINAN and SINASC).

**Table 1:** Missing data for the Brazilian Notifiable Diseases Information System, the Brazilian Live Birth Information System, and the Brazilian Mortality Information System, 2007–2012.

Databases	Records	Percentage missing		
		Maternal name	Maternal age	Municipality
Notifiable Diseases Information System SINAN)-notifications of dengue in women	1,725,943	0.4%	0.07%	0%
Live Birth Information System (SINASC)	17,387,267	0.03%	<0.01%	0%
Mortality Information System (SIM).- Stillbirths	187,487	2%	15%	0%
Mortality Information System (SIM).—Maternal Deaths	10,259	0%	18.5%	0%

### Ethics approval and consent to participate

We obtained ethical approval from the Research Ethics Committee, Public Health Institute, Federal University of Bahia, Brazil (CAAE: 26797814.7.0000.5030 CEP-ISC) and the London School of Hygiene and Tropical Medicine, UK (Ethics Ref: 10269).

### Linkage strategy

We derived our pre-processing and blocking schemes, gold-standard data and match weights based on previous linkage of Brazilian birth data, as described by Paixao et al (2017)[6]. First we prepared the data by excluding records without names and with generic names such as “ignorado, hospital name”, and deleting punctuation and extra spaces, and unknown prefixes. We transformed known abbreviations for names (e.g. Ap → Aparecida) into full names, and all names into upper case, dropping middle initials. We then blocked the records by municipality and used the Jaro-Winkler string comparator to calculate the similarity between names recorded in each dataset[12].

To estimate parameters for linkage weights and to validate the quality of the linkage, we created a gold-standard dataset. Firstly, we linked using deterministic linkage with exact agreement on full name and age. In subsequent steps we relaxed the rules by allowing matches with differences in age or in the ways names were recorded. Each step was followed by manual review to exclude false-matches[6].

Match weight calculations were based on the Fellegi-Sunter method[13]. For each record pair we calculated a probabilistic match weight based on two conditional probabilities: m-probability, P (agreement|match) and u-probability, P (agreement|non-match). We estimated m-probabilities for each identifier from the true matches in the gold-standard dataset. We calculated u-probabilities based on a list of non-matches, created from all pairwise comparisons of records within SINAN, excluding those belonging to the same individual.

Frequency-based weights were calculated for each category of Jaro-Winkler score comparator (for name of the woman) and year of age, so that rarer values were given higher weights. Separate weights were also calculated for the five most frequently occurring names in the data (Maria, Ana, Santos, Souza and Oliveira).

Since we were linking different subjects (the woman and her live or stillbirth), and because the exposure (dengue during pregnancy) could have happened up to nine months before the outcome (birth/ maternal death), there were timing issues to consider. Two records could differ in time by nine months and could bridge calendar years; some women would have a birthday between the date of dengue notification and the date of the live birth/death. To allow for this, we estimated different weights according to the similarity of age across datasets: equal ages, age differing by one year, age differing by two years, and ages differing by more than two years. In the maternal mortality linkage, we included the time between the birth outcome and death of the mother as a linkage variable (occurred on the same day as the birth outcome (day 0 or 1); between 2–7 days; between 8–15 days; between 16–30 days; after 30 days).

Match weights were calculated by summing the log of the ratio of m-probabilities and u-probabilities across different identifiers. The algorithm was implemented in Stata 14.1 and R 3.4.1.

Records pairs were ordered by match weight and manually inspected to identify a conservative threshold values aiming to exclude as many as false-matches as possible (prioritising a high positive predictive value). We classified any records above the cut-off threshold as links.

### Statistical analysis and evaluation of linkage quality

For the first linkage stage (of women with dengue notification to women with live births/stillbirths), we did not know the expected number of matches *a priori*, as we did not know how many pregnant women were expected to link with a dengue notification registered in SINAN. These findings are described by Paixao 2017[6].

For the second linkage stage (of maternal mortality), we expected all maternal deaths would link with a live birth or a stillbirth after excluding women with maternal deaths coded under pregnancy with abortive outcome (ICD-10 codes O00-O08; 10% of maternal mortality notifications). We therefore expected around 90% of maternal morbidity records to have a pregnancy outcome (stillbirth or live birth) and therefore to link with one of our datasets. We used this value to identify the number of missed matches (records from the same mother-baby pair that failed to link) and to estimate the sensitivity (true links among the matches) of the linkage.

To estimate the proportion of false matches (records that were erroneously linked from different mother-baby pairs), we looked at women who were coded as having died in a pregnancy with an abortive outcome (i.e. those who should not have linked with a live birth/stillbirth record). In Brazil, if women had an abortive outcome, the product of conception would not be recorded in either SINASC or SIM, therefore we did not expect a link from these women. Those who linked were assumed to be false-matches, as were those who linked to a live birth and a stillbirth simultaneously, unless they were multiple births.

We then examined which characteristics were associated with missed matches in the maternal mortality dataset. We compared the characteristics of maternal deaths classified as having dengue through linkage, with the maternal deaths where dengue was coded (using ICD-10 codes) as an underlying cause of death but that were missed by our linkage. We examined maternal age in years (continuous variable), maternal education (illiterate to 3 years of education, 4–7 years of education, more than 7 years or education), maternal marital status (married, divorced/widow or single), self-identified race (white, mixed/black, or other) and classification of deaths occurring during pregnancy or puerperium. Categorical variables were compared between groups with Chi^2^ test or Fisher’s exact test, and means were compared with a t-test. A two-sided P value of less than 0.05 was considered to indicate statistical significance. Stata version 14.1 was used for the statistical analyses.

## Results

### Maternal Mortality linkage

**Fig 2:**
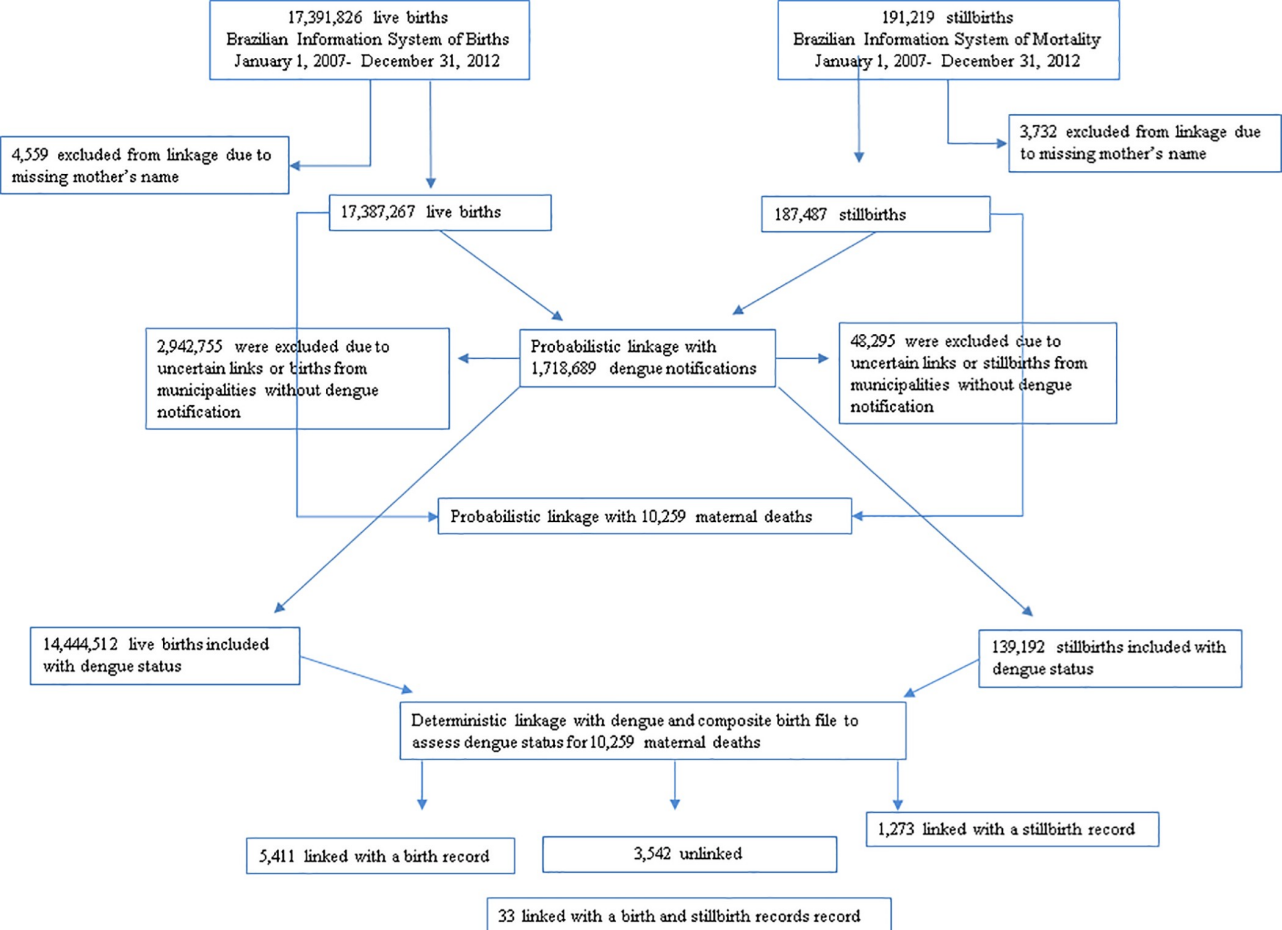
Number of records from Brazilian Information System of Notifiable Disease and Brazilian Information System of Mortality and Birth.

6717 of the 10,259 maternal deaths in SIM from 2007–2012 were linked: 5,444 (53%) linked to a live birth record, and 1,306 (13%) to a stillbirth record. Of these linked records, 33 linked to both a live and stillbirth simultaneously: 20 were multiple pregnancies (with a live birth and stillbirth outcome); 3 were duplicate records (stillbirths misreported as live births and notified in both datasets); and 10 were false matches. After excluding these, 3542 maternal deaths remained unlinked (Fig 2).

Of the 10,259 maternal deaths in SIM, 1,046 (10%) were coded as having an abortive outcome, which we did not expect to link to a live or stillbirth. Of these pregnancies with an abortive outcome, 124 (12%) linked (i.e. were false matches); 55 linked with a live birth and 71 with a stillbirth (two linked with both). This gave an estimated specificity of 922/1046 = 88% (Table 2). However, since misclassification of abortion and stillbirth is very common (with either a stillbirth being coded as an abortion or an abortion as a stillbirth), we also estimated the specificity for linkage with live births only, which was 991/1046 = 95%. After excluding the 1,046 maternal deaths with abortive outcomes from the 10,259 coded pregnancies, we expected that all the remaining 9,213 maternal death records would have linked with a live birth or stillbirth record. Instead, we observed only 6717 linked records, of which 124 were the aforementioned false matches and 6,593 were true links (Table 2). This gives an estimated sensitivity of 6,593/9213 = 72% and a positive predictive value (PPV) of 6593/6717 = 98%.

**Table 2:** Linkage accuracy.

	Match	Non-match	Total
**Link**	6593	124	*6717*
**Non-link**	2620	922	*3542*
Total	*9213*	*1046*	**10259**

### Maternal mortality and live births/stillbirths linkage errors

Missed matches were not associated with age or marital status (Table 3). Records were more likely to link if women had more than 7 years of education or self-declared as Caucasian. Records were 5.5 times more likely to link if maternal death occurred in the puerperium than if the death was classified as occurring during pregnancy.

**Table 3:** Associations between linkage accuracy and maternal characteristics.

	True matches N = 6593 n (%)	Missed-matches N = 2620 n (%)	OR(95%CI)	p-value
**Age of the mother in years**				
Mean age	28.4	28.7	-	0.127
			
**Marital status**				
Single	3,615 (54.8)	1,427 (54.5)	1	
Married	2,264 (34.3)	914 (34.9)	0.98(0.89–1.1)	
Divorced/widow	148 (2.3)	68 (2.6)	0.85 (0.64–1.1)	0.611
Missing	566 (8.6)	211 (8.0)		
**Maternal education**				
8 or more years	2,236 (33.9)	750 (28.6)	1	<0.001
4–7 years	1,773 (26.9)	699 (26.7)	0.85 (0.75–0.96)	
Less than 3 years	1,009 (15.3)	510 (19.5)	0.66 (0.58–0.76)	
Missing	1,575 (23.9)	661 (25.2)		
**Self-identified race**				
White	2,322 (35.2)	846 (32.3)	1	0.005
Mixed and black	3,787 (57.4)	1,583 (60.4)	0.87 (0.79–0.96)	
Others	91 (1.4)	51 (1.9)	0.65 (0.45–0.92)	
Missing	393 (6.0)	140 (5.3)		
**Period of Pregnancy**				
Pregnant	1,173 (17.8)	1,331 (50.8)	1	0.001
Puerperium	4,870 (73.9)	993 (37.9)	5.5 (5.0–6.1)	
Missing	550 (8.3)	296 (11.3)		

## Maternal Mortality linkage and dengue

We identified 23 maternal deaths that were coded in SIM with ICD-10 codes for dengue as the underlying cause of death, but that had not linked to the dengue notification cases in our linkage. The reasons were:

- the maternal death was in a pregnancy with an abortive outcome (the record should have been excluded from the previous linkage, n = 3 cases);
- the maternal death record did not link to a live birth or stillbirth (missed match, n = 8 cases);
- the maternal death linked with a live birth or stillbirth, but not to the dengue notifications (misclassification of dengue status, n = 12 cases).

We investigated these latter 12 cases further, finding that in 5/12 cases, the symptoms of dengue began in the puerperium, more than 42 days after the fetus was born, and therefore were classified as no dengue during pregnancy in the previous linkage; 1/12 cases were classified as an uncertain link; the remaining 6/12 were false-matches in the original linkage.

Excluding the three abortive outcomes, there were no differences in socio-demographic characteristics between the 20 records that our linkage failed to identify, and the 14 cases of dengue identified in our linkage of maternal mortality and dengue datasets.

## Discussion

We implemented the linkage methods described by Paixao et al (2017)[6] in a dataset with a known number of expected matches, and consequently were able to quantify measures of linkage accuracy and validate our previous linkage strategy. We therefore demonstrated that the quality of a particular linkage strategy can be validated using a dataset where the expected number of matches is known. Due to the lack of common variables for linkage, it was not possible to increase the sensitivity without increasing the number of false matches. However, our linkage achieved a PPV of 98%, indicating that the majority of links were correct. In our evaluation of linkage error, we observed that mothers with fewer years of education and mixed ethnic background were more likely to be missed from the linkage. However, there were no differences in the socio-demographic characteristics between the cases of dengue identified in our linked dataset, and those that were missed.

Linkage to bring together information from two different sources about the same individual can achieve high rates of sensitivity, even when using records containing truncated or ambiguous matching variables[14–16]. However, bringing together information from two different people (in this case, mother and baby) has been considered to be a more difficult task, due to a lack of common variables. Sensitivity in these cases tends to be lower, e.g. 38% missed links in Georgia[17] and 20% in New Jersey[18]; our results (28% missed records) are within these ranges. Specificity in our study was estimated at between 88% (pregnancies with an abortive outcome that linked with either live or stillbirths) and 95% (pregnancies with an abortive outcome that linked with live births only), but it was difficult to distinguish between false matches and misclassification of stillbirths/abortive outcomes or a maternal death falsely recorded as being caused by (or associated with) an abortion or an abortive outcome.

A very important aspect of linkage is how linkage error might impact on inferences from the linked dataset. The analyst should know and report any evaluation of linkage accuracy and be aware of groups disproportionately affected by linkage error [19,20]. In this study, we found differences between the matches and the residual (non-linked) records: the matches were more likely to have 7 or fewer years of education or self-declare as non-Caucasian compared with the non-links; both these characteristics reflect social disadvantage. Adams et al in USA found that incomplete records were related to social disadvantage and consequently these records were less likely to match[17].

Beyond socioeconomic status, there are other possible explanations for the distinctive characteristics observed in the non-linked group. First, completion of the forms by health care professionals and the process of digitalization in private and public health facilities may result in differential quality of records. Second, in Brazil, unsafe induced abortion has been associated with low education and income[21,22], and because induced abortion is illegal for most indications, it may be that some missed maternal death matches had an abortive outcome resulting from an illegal and unsafe abortion, which were coded under a different cause of death in the legal document. Therefore the missed-matches may not all be linkage errors but coding errors, which would explain the differences observed between these two groups. The fact that most of the missed-matches occurred during pregnancy rather than postpartum supports this hypothesis.

To further evaluate the linkage accuracy on the dengue status of the pregnant women (exposure variable), we compared the dengue cases that the algorithm captured, with those that it could not (i.e., those identified in post-hoc analyses as individuals coded with dengue as cause of death). We found no difference in socio-demographic characteristics between the two groups. It is therefore unlikely that the linkage process introduced bias in our previous linkage of live births/stillbirths and dengue, since missed matches occurred randomly in relation to the exposure variable. However, this analysis had limited power because the sample size was small (34 cases of dengue). Therefore, we interpret our results showing no difference between the two groups cautiously.

This study has a number of limitations. First, maternal deaths are under-reported. However, as this error probably affected both those exposed and not exposed to dengue, it is unlikely to bias the study results. Second, we did not know the gestational age at which each maternal death occurred, and so we cannot conclude whether the missed-matches occurred due to linkage errors, or if they should not have been linked (i.e., had abortive outcomes). It is possible that we underestimated the number of false-matches, but we expected all the maternal deaths to link, it is unlikely that records have liked to the wrong record, because that record would also have been linked. Due to the restricted number of variables, we could not improve the sensitivity of the algorithm, and so our linked dataset should not be used to estimate rates of live births or maternal mortality rates for women exposed to dengue during pregnancy. Our analysis of the association between study characteristics and linkage error may have been limited due to low power. Finally, we assumed that linkage quality metrics in our maternal mortality linkage would be representative of those in our initial linkage between birth outcomes and dengue notifications, since the data are derived from similar sources and contain the same matching variables.

It is important to understand the quality of the linkage and the potential bias that can be introduced in results of analyses of linked data. In this study, sensitivity and specificity of our linkage strategy were comparable with previous literature. Although there were no differences in the characteristics of dengue cases missed or included in our linked dataset, linkage error occurred disproportionately according to some social-demographic characteristics, which introduces bias and should be taken into account in future analyses. These results reinforce the need to evaluate linkage quality and to take linkage error into account within analyses of the following studies when necessary.
